# UPR-induced ovarian cancer cell fusion: a mechanism favoring drug resistance?

**DOI:** 10.18632/oncoscience.575

**Published:** 2023-05-11

**Authors:** Melisa Husein, Patrick Petignat, Marie Cohen

**Keywords:** ovarian cancer, polyploidy giant cancer cell, unfolded protein response, drug resistance

Epithelial ovarian cancer is an almost universally fatal disease, ranked as the top cause of gynecologic cancer-related deaths in industrialized countries in 2021. Apart from most diagnoses being advanced-stage diseases, the recurrence rates are exceptionally 70% within the first five years. In addition to surgical resurrection, the overall primary treatment for cancer is adjuvant care with chemotherapeutic drugs such as paclitaxel in combination with platinum-based reagents. Recurring ovarian cancers are associated with chemotherapy resistance and high death rates because of resistance to provided therapies. In this editorial, chemotherapeutic resistance in recurring ovarian cancer will be discussed with a focus on the unfolded protein response (UPR) and its effect on polyploid giant cancer cells (PGCCs) formation.

The importance of understanding recurring and chemotherapy-resistant ovarian cancers includes investigating cancer cell molecular mechanisms. Related to this, a quiescent population of cancer cells has been in the spotlight. Cancer stem cells represent a small percentage of the total cancer cells and due to their pluripotent properties induce recurrent cancer, metastasis, and chemotherapeutic resistance. Zhang et al. revealed PGCCs share similarities with cancer stem cells. The giant cells derived from an ovarian cancer cell line, SKOV3, were in accordance with our previous results [1] shown to be, at least partially, formed by cell fusion. PGCCs were identified as stress-induced cancer cells and in comparison, to normal ovarian cancer cells, PGCCs have increased tumorigenicity. The gained properties of PGCCs favor not only tumor growth and heterogenicity but chemotherapeutic therapy resistance [2]. These ovarian PGCCs are mainly found in the hypoxic microenvironment of tumor tissue [3].

The growing tumor forms a dysfunctional vascularization with insufficient blood supply, resulting in a hypoxic tumor microenvironment. As performed in the study of Zhang et al., mimicking these hypoxic conditions *in vitro* selects for PGCC growth in a heterogeneous cancer cell population [2]. The cellular response to hypoxia involves an elevated expression of specific hypoxia-induced transcription factors, due to the cellular adaptive response in both malignant and benign tumors [4]. These molecular changes affect endoplasmic reticulum (ER) homeostasis and result in dysregulation in the posttranslational folding and secretion of proteins. Hypoxia is far from the only cause of ER stress. High metabolic demand, dysregulated translational and transcriptional processes, and cytotoxic drug therapies are numerous additional processes inducing ER stress. These processes are relatable to cancer cell demands and cancer treatment. As the downstream effect of the ER stress response (or unfolded protein response, UPR) influences cell fate, its activation in the tumor environment will cause cancer cell adaption to apoptosis and chemotherapeutic agents [5].

The ER chaperone protein glucose-related protein 78 (GRP78) is the primary marker of UPR activation [5]. Its expression has been related to ovarian cancer [6, 7] and resistance to apoptosis [8, 9]. In addition, it was shown that the inhibition of the inositol-required enzyme 1α (IRE1α) branch of the UPR may enhance the efficacy of chemotherapy in ovarian cancer [10, 11].

Taking advantage of two ovarian cancer cell populations expressing different nuclear fluorescent proteins and different antibiotic resistance genes, Yart et al. evaluated the role of UPR on ovarian PGCC formation and chemotherapeutic resistance. They demonstrated that the generation of ovarian PGCCs may be a result of ovarian cancer cell fusion, which could be promoted by the chemotherapeutic drug, Paclitaxel, and/or the UPR. The derived hybrid cells displayed a single nucleus with genetic material from both parental cells. They restarted a cell cycle and retained similar proliferative and tumorigenic capacities as their parental cells. In addition, the hybrid cells acquired double antibiotic resistance from their parental cells, and their invasiveness properties were increased compared to their parental cells [1]. This mechanism could be transposed to ovarian cancer cells exposed to paclitaxel treatment. Paclitaxel treatment induces UPR, which enhances ovarian PGCC formation. Ovarian PGCCs are dormant cells, which are no longer affected by drugs targeting dividing cells such as paclitaxel. When conditions become propitious, these cells may mix their genetic material, become more genetically unstable and proliferate again with new tumorigenic properties.

In summary, more and more evidence shows that the ovarian PGCCs are, at least partially, formed by cell fusion and have the capacity to proliferate and promote tumor formation as well as resistance to chemotherapy. The tumor’s hypoxic conditions and chemotherapeutic treatment may induce UPR and consequently the formation of ovarian PGCC with properties of chemotherapeutic resistance. Altogether, the findings suggest that the UPR modulation in combination with conventional chemotherapeutic drug treatments of ovarian cancer patients could represent an interesting therapeutic strategy to avoid the formation of PGCCs and therefore limit cancer relapse, and drug resistance.
